# Case Report: Single-incision robotic-assisted nephrectomy and heterotopic renal autotransplantation for a large complex renal artery aneurysm

**DOI:** 10.3389/fsurg.2026.1848812

**Published:** 2026-08-14

**Authors:** Matthew Gaynor, Mahmoud Morsi, Javier Gonzalez, Omaida Velazquez, Henry Ocando Nava, Gaetano Ciancio

**Affiliations:** 1 Department of Surgery, University of Miami Miller School of Medicine, Miami, FL, United States; 2 Miami Transplant Institute, University of Miami Miller School of Medicine, Miami, FL, United States; 3 Servicio de Urología, Unidad de Transplante Renal, Hospital General Universitario Gregorio Marañón, Madrid, Spain; 4 Division of Vascular and Endovascular Surgery, University of Miami Miller School of Medicine, Miami, FL, United States; 5 Department of Urology, University of Miami Miller School of Medicine, Miami, FL, United States; 6 Jackson Memorial Hospital, Miami, FL, United States

**Keywords:** complex vascular reconstruction, *ex vivo* aneurysm repair, minimally invasive surgery, renal artery aneurysm, renal autotransplantation, renovascular hypertension, robotic-assisted nephrectomy

## Abstract

Renal artery aneurysms (RAAs) may require complex open vascular surgery. We describe a 50-year-old female with a large, intricate right RAA involving multiple branches. Management consisted of single-incision robotic-assisted right nephrectomy, *ex vivo* aneurysm excision, and arterial and venous reconstruction with heterotopic kidney autotransplantation to the right iliac fossa. Following reperfusion, partial thrombosis of the reconstructed branches occurred, which was successfully revised using reconstruction with the patient's right internal iliac artery. Postoperatively, kidney function was preserved, and no complications developed. *Ex vivo* repair with heterotopic kidney autotransplantation may represent a safe and effective strategy in selected patients with complex RAAs.

## Introduction

Renal artery aneurysm (RAA) is a rare condition, though it is encountered more frequently in tertiary referral centers. Reported prevalence estimates range from 0.3 to 1.3% in the general adult population (1–3). Patients with a RAA may present with a variety of symptoms, including hypertension, renal ischemia, hematuria, and rarely rupture (4). However, most patients with a RAA are asymptomatic, and these RAAs are often diagnosed incidentally during imaging performed for unrelated complaints. Several reports have noted the limited utility of clinical symptoms in establishing a diagnosis (5). According to the Society for Vascular Surgery 2020 clinical practice guidelines on the management of visceral aneurysms, indications for intervention in RAA include: symptomatic aneurysm, aneurysm size >3 cm, women of childbearing age, refractory hypertension, and enlarging aneurysm (6).

Management options for RAAs include open surgical repair, such as aneurysmorraphy, as well as endovascular techniques, including coil embolization and stent grafting (1–5). Advances in minimally invasive surgery have led to the increasing use of robotic-assisted approaches in select cases (7). In selected cases, particularly when aneurysms are small or asymptomatic, a surveillance protocol may be appropriate (1–3). However, management of large, complex RAAs involving multiple distal branches remains particularly challenging, as both endovascular and *in situ* minimally invasive techniques are often limited by unfavorable vascular anatomy (4, 5). In these scenarios, nephrectomy with *ex vivo* repair and subsequent autotransplantation may provide a viable alternative, though reports combining this approach with single-incision robotic-assisted nephrectomy (RAN) remain limited (7, 8). Here, we present a case demonstrating the feasibility and effectiveness of our clinic's approach: RAN followed by *ex vivo* aneurysm repair and heterotopic renal autotransplantation for the management of a large, complex, branch-involving RAA (9). It should be noted that the novelty of our approach does not lie in the individual components of the procedure (i.e., RAN, *ex vivo* renal artery aneurysm repair, and heterotopic renal autotransplantation), but rather in the vascular reconstruction strategy used to salvage and preserve a complex hilar renal artery anatomy following aneurysm excision.

## Case report

A 50-year-old woman with a long-standing history of hypertension (for nearly 30 years) was referred for evaluation of bilateral renal artery aneurysms in 2025. The patient required multiple antihypertensive medications, including atenolol (50 mg BID), losartan (100 mg daily), and hydrochlorothiazide (25 mg daily) for adequate blood pressure control. She also had a prior cerebrovascular accident in 2015 without lasting neurologic sequelae. The diagnosis was established by computed tomography angiography (CTA), which revealed three distinct RAAs involving both kidneys. CTA demonstrated two right-sided aneurysms located in close proximity to each other. The aneurysms measured approximately 3.0 × 2.2 cm and 2.5 × 2.0 cm, respectively. The larger lesion exhibited peripheral wall calcification, whereas the second aneurysm demonstrated partial intraluminal thrombosis. The left-sided aneurysm was still sizeable, at approximately 1.3 × 1.0 cm, but appeared less severe than those on the right. CTA also demonstrated features of fibromuscular dysplasia; however, the proximal renal arteries were widely patent. Given the size, morphology, and complexity of the right-sided aneurysms, surgical intervention was recommended.

The proposed treatment for this case was approved by the University of Miami Institutional Review Board and conducted in accordance with the ethical principles of the Declaration of Helsinki, as revised in 2013. The multidisciplinary team consisted of vascular and transplant surgeons.

## Methods (surgical technique)

### Robotic-assisted right nephrectomy

#### Patient positioning and preparation

**Figure 1:**
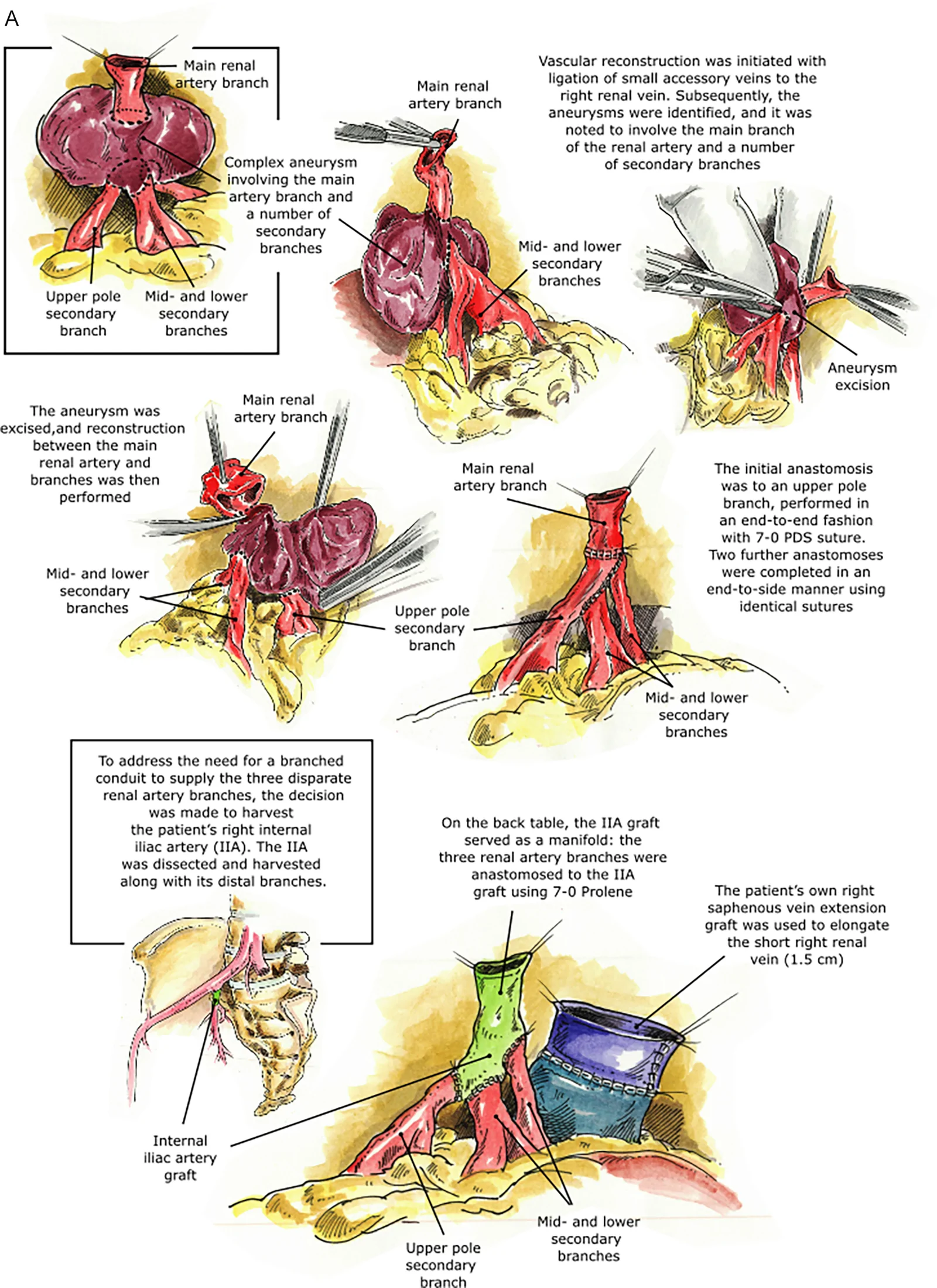

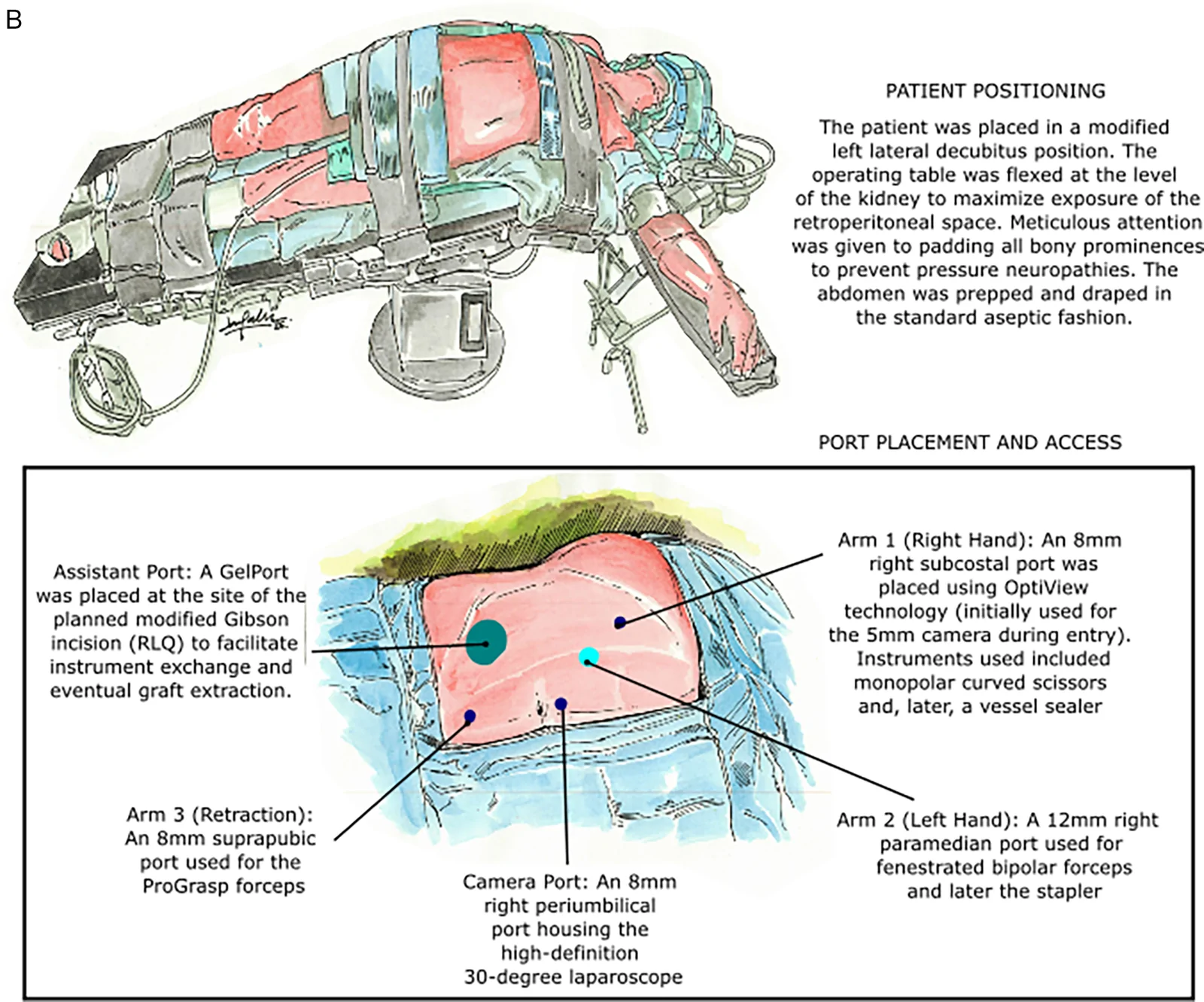
**(A)** Drawing of the different steps from resection of the right renal aneurysm, including the first right renal artery reconstruction and the second vascular reconstruction using the patient's own right internal iliac artery and the placement of the patient's own right saphenous vein extension. **(B)** Image of patient position and ports placement for robotic-assisted right donor nephrectomy.

The patient was placed in a modified left lateral decubitus position (Figure 1A). The operating table was flexed at the level of the kidney to maximize exposure of the retroperitoneal space.

Meticulous attention was given to padding all bony prominences to prevent pressure neuropathies. The abdomen was prepped and draped in the standard aseptic fashion. A surgical timeout was performed to verify patient identity and procedural specifics.

#### Port placement and access

Pneumoperitoneum was established using Palmer's technique via a Veress needle inserted in the right upper quadrant. Robotic ports were placed under direct vision along the midclavicular line (Figure 1B).

1. **Camera Port:** An 8.0 mm right periumbilical port housing the high-definition 30-degree laparoscope.
2. **Arm 1 (Right Hand):** An 8.0 mm right subcostal port was placed using OptiView technology, which was initially used for the 5.0 mm camera during entry. Instruments used included monopolar curved scissors and later, a vessel sealer.
3. **Arm 2 (Left Hand):** A 12.0 mm right paramedian port used for fenestrated bipolar forceps and later, the stapler.
4. **Arm 3 (Retraction):** An 8.0 mm suprapubic port used for the ProGrasp forceps.
5. **Assistant Port:** A wound retractor system (GelPort; Applied Medical, Rancho Santa Margarita, CA, USA) was placed at the site of the planned modified Gibson incision (RLQ) to facilitate instrument exchange and eventual graft extraction.

#### Robotic dissection

With the system docked, the right colon was mobilized medially to expose the retroperitoneum. The hepato-renal ligament was divided to release the upper pole of the kidney, and a laparoscopic grasper was utilized for liver retraction.

We proceeded to dissect the right gonadal vein and the renal vein. The ureter was identified and isolated down to the level of the right common iliac artery, preserving the peri-ureteral vascular plexus. Using the fourth robotic arm (ProGrasp) for elevation and traction, we gained clear access to the renal hilum. The right adrenal gland was carefully dissected away from the upper pole of the kidney and preserved. Following this, the patient's fascia was dissected posteriorly to fully liberate the kidney.

#### Vascular control and nephrectomy

Attention was turned to the renal hilum. The renal vein was fully skeletonized. Behind the vein, the arterial anatomy was dissected. We identified a single main renal artery trunk that trifurcated into three branches distally, corresponding to the complex aneurysmal anatomy seen on imaging. The dissection was carried proximally toward the aorta to ensure the stapler would encompass the main trunk proximal to the aneurysms and the trifurcation.

The ureter was clipped distally and transected. While the kidney was elevated using robotic arms 1 and 4, a robotic stapler (SureForm 45 mm; Intuitive Surgical, Sunnyvale, CA, USA) was introduced through the 12.0 mm port on arm 3. The single main renal artery trunk was stapled and divided. Subsequently, the renal vein was stapled and divided.

#### Graft extraction

The kidney was immediately placed into a Laparoscopic Specimen Retrieval System bag. The graft was extracted through the GelPort incision in the right lower quadrant and handed to the back-table team for immediate flushing and reconstruction. The robotic system was undocked.

Hemostasis was confirmed in the renal fossa, and the port sites were closed in the standard fashion.

### *Ex vivo* repair of aneurysm

The robotic-assisted procured right kidney was prepared on the back-table after being flushed with histidine–tryptophan–ketoglutarate solution (HTK; Essential Pharmaceuticals, Durham, NC, USA) until fluids from the right renal vein were clear. Perinephric fat was trimmed, and a lower biopsy was performed, which was closed with 5-0 polydioxanone suture (PDS; Ethicon, Somerville, NJ, USA) (10, 11). The renal artery, with a large complex RAA, and renal vein were identified and dissected. The ureter was then identified and dissected.

**Figure 2:**
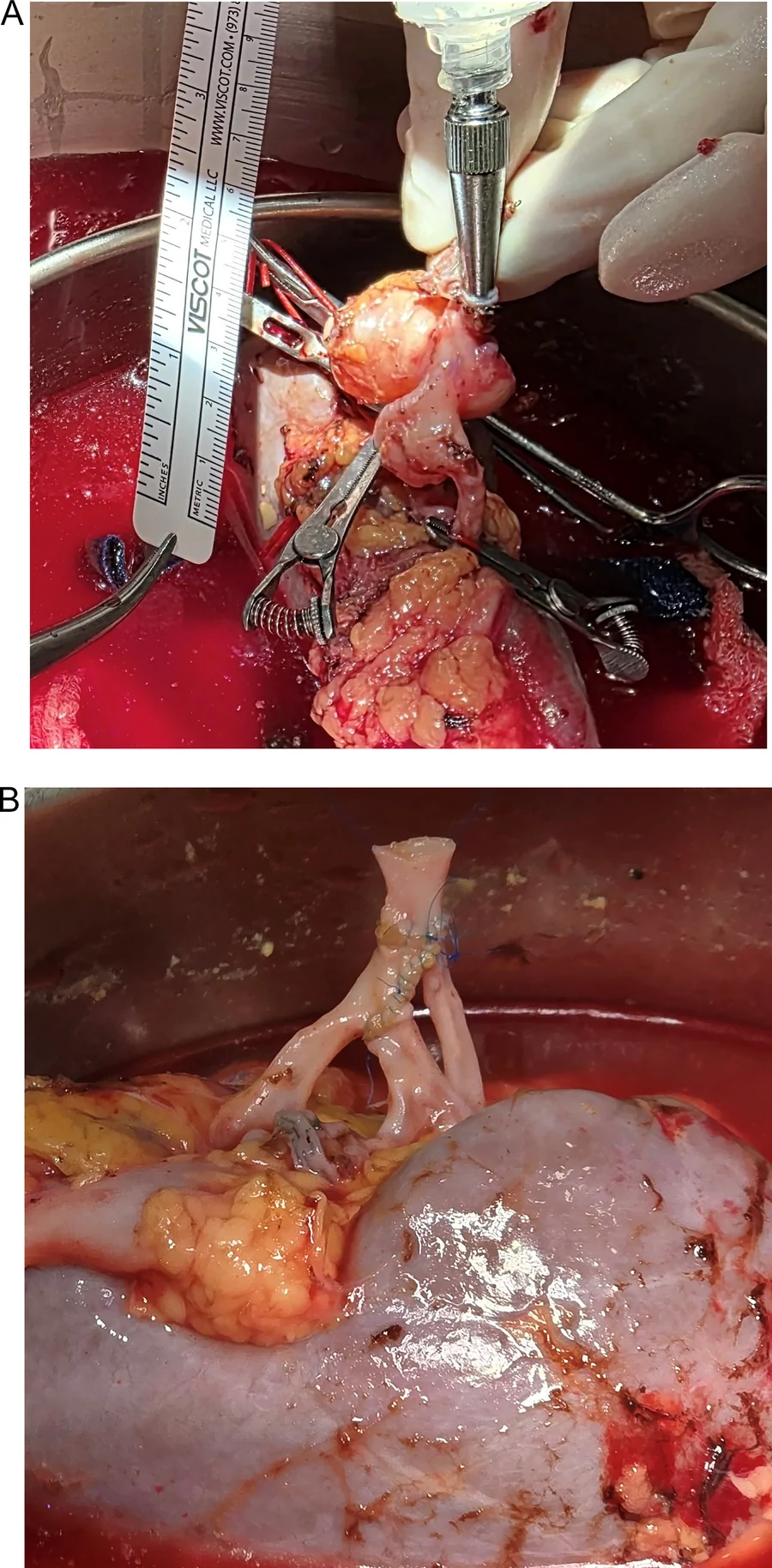
**(A)** Saccular aneurysm of the right renal artery. **(B)** Vascular reconstruction after resection of the two right renal artery aneurysms.

The patient's vascular reconstruction was initiated with ligation of small accessory veins to the right renal vein. Subsequently, the aneurysms were identified and found to involve the main renal artery as well as numerous secondary branches (Figure 2A). The aneurysms were excised, followed by reconstruction of the main renal artery and its branches (Figure 2B). The initial anastomosis was performed to an upper pole branch, performed in an end-to-end fashion with 7-0 PDS suture. Two further anastomoses were then completed in an end-to-side manner using the same suture material. The patient's right saphenous vein was used as an extension graft to lengthen the short right renal vein (approximately 1.5 cm).

### Heterotopic autotransplantation

Similar to kidney allograft placement in renal transplant recipients, the heterotopic autotransplant was positioned in the right iliac fossa. The previously created right lower quadrant incision for the removal of the right kidney was utilized for the autotransplant. The inferior epigastric vessels were identified, ligated, sutured with 2-0 silk, and divided; the round ligament was likewise ligated with 2-0 silk and divided. A Bookwalter retractor was then placed. The parietal peritoneum, which had been opened during the kidney retrieval, was then closed and carefully dissected off the abdominal wall to create a 1.0 cm flap. Limited dissection of the lymphatic and areolar tissue surrounding the external iliac artery and vein was performed, providing sufficient space for placement of vascular clamps.

The external iliac vein was clamped with LambertKay (LambertKay; V. Mueller, Chicago, IL, USA) and Fibra spring clips (FibraSpring; Teleflex, Morrisville, NC, USA). G-1250 12.0 mm soft vascular clamps were used to occlude the external iliac artery (7). The external iliac artery was anastomosed first by continuous suture using 7-0 polypropylene suture (Prolene; Ethicon, Somerville, NJ, USA). The external iliac vein anastomosis was then performed with a 5-0 polypropylene suture. The ureter was trimmed to prevent rotation or kinking, allowing for a tension-free anastomosis to the mobilized bladder. An extravesical ureteral anastomosis was performed using our previously described ureteral reimplantation technique (7).

## Results

Total robotic-assisted right nephrectomy time was two hours with minimal blood loss of 150 mL. During the initial procedure, total warm ischemia time was 19 minutes, and cold ischemia time was 180 minutes. Upon reperfusion, the kidney remained soft with patches of ischemia, which turned the kidney partially blue. Doppler signals were absent in the distal poles. Diagnosis of arterial inflow failure was suspected, likely due to dissection/thrombosis. The kidney was immediately explanted and flushed with HTK.

Partial thrombosis was identified in two of the three branches of the main renal artery. In addition, the kidney endothelium had been compromised as a result of the prior dissection. Given these findings, we concluded that the previous reconstruction was deemed non-viable. Because revascularization required a branched conduit to supply the three renal artery branches, the decision was made to procure the patient's right internal iliac artery (IIA). The IIA was carefully dissected and procured with its intact branches. On the back table, the IIA graft was fashioned as a manifold, with each of the three renal artery branches anastomosed to IIA branches using 7-0 Prolene sutures (Figure 2B).

The proximal end of the IIA graft was then anastomosed to the right external iliac artery (EIA). Upon releasing the clamps, the kidney perfused immediately, becoming firm and pink with excellent turgor. Palpable pulses were present throughout the graft. Total warm ischemia time was minimized despite the revision. A new ureteroneocystostomy was performed to restore urinary continuity. During the revision, total warm ischemia time was 25 minutes, and cold ischemia time was 35 minutes.

Postoperatively, the kidney immediately started making urine, and renal function remained stable. The patient was in the hospital for five days, and she was discharged with a serum creatinine of 1.0 mg/dL. At postoperative follow-up, the patient demonstrated preserved right kidney function without complications. A surveillance kidney ultrasound showed good flow for both the renal artery and renal vein at 3 months after surgery, with a creatinine of 1.1 mg/dL. The patient will continue her follow-up with the vascular surgery team and her primary care physician. She also continued with the same blood pressure control as she had been receiving prior to (and at the time of) her RAA presentation: atenolol (50 mg BID), losartan (100 mg daily), and hydrochlorothiazide (25 mg daily).

In terms of considering alternative approaches, as described earlier, the patient's renal artery aneurysm was located at the renal hilum and involved bifurcation/trifurcation of the main renal artery with multiple segmental branch vessels originating directly from the aneurysm sac (Figure 2B). Preoperative CTA with three-dimensional reconstruction demonstrated inadequate proximal and distal landing zones for endovascular stent-graft placement. Therefore, deployment of a covered stent would have necessarily excluded one or more major segmental branches, resulting in substantial kidney parenchymal ischemia and loss of kidney function. Coil embolization was similarly not feasible, because the aneurysm lacked a discrete neck and incorporated multiple branch vessels arising from the aneurysm wall. Embolization would have carried a high risk of occluding these branches and causing significant kidney infarction.

In-situ open reconstruction was also considered but was deemed technically unfavorable due to the complex hilar anatomy, deep retroperitoneal location, involvement of multiple second-order branches, and the anticipated prolonged warm ischemia time required for meticulous branch reconstruction. Given the complexity of the vascular anatomy, *ex vivo* repair on the back table provided superior exposure, allowed precise reconstruction of all segmental branches under cold preservation, minimized ischemic injury, and maximized preservation of kidney function.

## Discussion

This case illustrates the difficulties in managing large, complex RAAs involving multiple distal branches. In such cases, conventional endovascular or *in situ* repair techniques are often not feasible (4, 5). Due to the aneurysm size, morphology, and branch involvement, a more complex reconstructive strategy was needed. Our clinic's approach, combining robotic-assisted nephrectomy with *ex vivo* repair and heterotopic renal autotransplantation, provides a safe and effective solution in carefully selected patients.

We acknowledge that robotic donor nephrectomy, *ex vivo* renal artery aneurysm repair, and kidney autotransplantation have each been previously described. The novelty of our approach does not lie in the individual components of the procedure, but rather in the vascular reconstruction strategy used to salvage and preserve a complex hilar renal artery anatomy following aneurysm excision.

Following aneurysm resection, reconstruction options considered included primary repair, direct branch reimplantation, autologous vein graft interposition (great saphenous vein), synthetic vascular graft, and arterial autografts. Primary repair and direct branch reimplantation were performed but resulted in thrombosis of the reconstruction. The saphenous vein graft was used to increase the length of the short right renal vein. Synthetic grafts were considered less desirable because of the small caliber vessels and potential thrombotic complications.

We selected the internal iliac artery as a salvage conduit because it provided an autologous arterial graft with a caliber closely matching the renal artery branches and a natural bifurcation that could be tailored to reconstruct multiple segmental vessels. The arterial wall characteristics, branching configuration, and excellent handling properties facilitated a tension-free reconstruction while preserving perfusion to all kidney segments. In our case, the internal iliac artery allowed anatomical restoration of the renal artery tree that would have been difficult to achieve with vein grafts or synthetic conduits.

Therefore, we believe the innovative aspect of this report is the combination of robotic nephrectomy, *ex vivo* aneurysm excision, using the same incision to both remove the kidney and to perform the autotransplant, and tailored internal iliac artery salvage reconstruction to preserve complex hilar vascular anatomy before autotransplantation.

The episode of early arterial thrombosis represented a critical learning point in this case. Although the precise etiology cannot be determined with certainty, several factors likely contributed. First, extensive vascular manipulation during *ex vivo* aneurysm resection and reconstruction may have resulted in endothelial or intimal injury, predisposing the vessels to thrombosis. Second, the complexity of the initial reconstruction involving multiple branch vessels may have created areas of turbulence, altered flow dynamics, or relative stenosis at the anastomosis sites. Third, vessels redundancy, torsion, or subtle kinking after graft reperfusion and positing within the iliac fossa may have compromised arterial inflow despite an initially satisfactory appearance.

Careful reassessment of the reconstruction demonstrated that optimization of the arterial geometry was necessary to improve flow characteristics and minimize the risk of recurrent thrombosis. The revision procedure focused on creating a more anatomical and tension-free configuration while ensuring adequate caliber vessels and alignment. All of that was achieved with the use of IIA. In cases involving multiple branch reconstructions, even minor technical imperfections may significantly affect flow dynamics and increase the risk of early graft thrombosis.

Our treatment approach is best understood in the context of the evolution of diagnostic and management strategies for RAAs. Historically, initial evaluation involved ultrasonography or intravenous pyelography, with angiography serving as the diagnostic gold standard (10). With the evolution of endovascular techniques, angiography is now primarily utilized as a therapeutic modality rather than a standalone diagnostic test. Moreover, combined diagnostic-therapeutic angiography is performed infrequently. Currently, CTA and magnetic resonance angiography (MRA) offer high sensitivity and specificity for diagnosing RAAs and are routinely used for both preoperative assessment and post-intervention surveillance (11).

Our patient initially underwent an abdominal ultrasound for evaluation of abdominal pain. She had a long-standing history of hypertension. Subsequent CTA evaluation demonstrated bilateral RAAs. These aneurysms were large and complex, involving multiple branches of the renal artery. The two right-sided aneurysms were contiguous, measuring approximately 3.0 × 2.2 cm and 2.5 × 2.0 cm, respectively. Additionally, there was a smaller left-sided aneurysm measuring approximately 1.3 × 1.0 cm. The severity of the left-sided aneurysm was not life-threatening, unlike the two right-sided aneurysms, so it was not removed during this particular procedure. Instead, the left-sided aneurysm is being managed with continuous surveillance and blood pressure control medication.

The decision to intervene in patients with RAAs is guided by aneurysm size, extent/complexity of multiple arterial branch involvement, symptomatology, risk of rupture, and patient-specific factors. Early historical studies emphasized the unpredictable behavior of RAAs and the potentially catastrophic consequences of rupture (1–3). Although many RAAs remain asymptomatic, surgical intervention is recommended for aneurysms greater than 3.0 cm in diameter, symptomatic aneurysms, aneurysms associated with hypertension or renal ischemia, and RAAs in women of childbearing age due to increased rupture risk during pregnancy (4, 5).

While surveillance may be appropriate for small, asymptomatic aneurysms, the threshold for intervention is lower in patients with progressive enlargement, complex anatomy, or refractory hypertension (5). The single patient in our study had long-standing hypertension and a large, complex aneurysm involving multiple arterial branches. While RAA complications are rare, they do occur. Some of the most notable complications are flank pain, hematuria, renal infarction, renovascular hypertension, and rupture (4). Early studies emphasized the association between rupture and significant consequential morbidity and mortality (1, 5). Therefore, surgical intervention was the favored option in our case.

Renovascular hypertension is one of the most common clinical manifestations of RAAs. This type of hypertension may originate from turbulent blood flow, distal embolization, or renal ischemia (5). Martin et al. (5) studied 39 patients and reported on selective hypertension treatments to prevent RAA rupture. The study demonstrated improvement or resolution of hypertension following aneurysm repair, which supports a causal relationship between RAAs and secondary hypertension (5). Martin et al. (5) emphasized selective surgical intervention not only to prevent rupture but also to achieve meaningful blood pressure control in affected patients (5). Patients who have undergone surgical intervention were determined to have been at high risk of developing a rupture. In our case, the patient's prolonged history of hypertension contributed to the decision to pursue surgical management.

For this patient's case, a robotic-assisted approach was utilized to facilitate nephrectomy before *ex vivo* repair and heterotopic renal autotransplantation. Over the last several decades, the advancement in minimally invasive surgery has led to increasing use of laparoscopic and robotic-assisted approaches for renal surgery (9). These techniques offer reduced postoperative pain, shorter hospital stays, and faster recovery compared to open surgery (12). Robotic platforms provide enhanced dexterity and visualization, facilitating precise vascular dissection and reconstruction in select patients.

Generally, minimally invasive *in situ* repair is limited to aneurysms with a favorable anatomy. An example would be saccular aneurysms involving the main renal artery without extensive branch involvement (13). Complex aneurysms that affect multiple segmental branches are challenging to treat using purely minimally invasive or endovascular techniques (4, 5). An endovascular or *in situ* repair approach was not suitable here given the complexity of the patient's anatomy.

Despite the use of RAN, the reconstructive portion of our procedure was performed *ex vivo* and required an open vascular procedure. The aneurysm's involvement of multiple distal branches precluded endovascular repair and made *in situ* reconstruction unfeasible. *Ex vivo* repair is particularly useful for safely removing renal artery aneurysms with extensive branch involvement. In this case, *ex vivo* repair provided the best visualization to remove the RAAs and perform reconstructive surgery on the renal artery and its branches. The primary challenge during the complex vascular reconstruction was the presence of renal artery fibrodysplasia, which was likely responsible for the thrombosis that developed after the initial revascularization.

After complete resection of all visibly diseased arterial endothelium, the renal artery was reconstructed using the patient's own right internal iliac artery. IIA grafts are advantageous because the tissue is flexible and matches the length needed to fully repair the renal artery (7). Following this reconstruction, the second reperfusion was successful (13).

Heterotopic renal autotransplantation has been used to remove RAAs safely (4). This transplant type allows for meticulous back-table vascular reconstruction under optimal visualization and cold ischemia. Using this technique minimizes injury to the kidney while permitting complex arterial and venous repairs (7, 8). Additionally, heterotopic implantation in the iliac fossa enables straightforward vascular anastomoses and facilitates postoperative monitoring (4). Ultimately, this technique, with the combination of RAN and open *ex vivo* reconstruction, offers a well-tolerated, durable (with a greater likelihood of the final surgical result being successful), and reproducible solution for patients with complex, branch-involving anatomy.

One important study limitation was the fact that while a 3-month CTA with three-dimensional reconstruction would have provided additional anatomical detail, further contrast-enhanced imaging was not available during this patient's postoperative follow-up. Consequently, surveillance was performed using serial Doppler ultrasonography, which demonstrated sustained graft perfusion, patent vascular reconstruction, and preserved kidney function without evidence of vascular complications.

## Conclusion

RAAs are rare complications. There is a significant risk for rupture if RAAs form in patients associated with complex anatomy or who have a long-standing history of hypertension (in this case, the involvement in multiple renal artery branches as well as the likely presence of extensive renal artery fibrodysplasia in which the diseased tissue needed to be removed). While minimally invasive and endovascular techniques are continuously evolving, they remain limited for managing large, branch-involving aneurysms. This case suggests that single-incision RAN followed by *ex vivo* aneurysm repair and heterotopic renal autotransplantation may be a safe and effective strategy for treating complex RAAs. This approach may be feasible in selected patients with complex hilar renal artery aneurysms, but it does require experienced multidisciplinary teams. Longer follow-up of this patient is also needed to assess durability. In experienced centers, this approach enables precise vascular reconstruction, preserves renal function, and may yield excellent clinical outcomes in carefully selected patients, including situations where back table reconstruction needs to be re-performed.

## Data Availability

The original contributions presented in the study are included in the article/Supplementary Material, further inquiries can be directed to the corresponding author.
